# Blue Light and Green Light Fundus Autofluorescence, Complementary to Optical Coherence Tomography, in Age-Related Macular Degeneration Evaluation

**DOI:** 10.3390/diagnostics15131688

**Published:** 2025-07-02

**Authors:** Antonia-Elena Ranetti, Horia Tudor Stanca, Mihnea Munteanu, Raluca Bievel Radulescu, Simona Stanca

**Affiliations:** 1 Doctoral School, “Carol Davila” University of Medicine and Pharmacy, Strada Dionisie Lupu No. 37, 020021 București, Romania; 2 Clinical Department of Ophthalmology, “Carol Davila” University of Medicine and Pharmacy, 020021 Bucharest, Romania; 3 Department of Ophthalmology, “Victor Babes” University of Medicine and Pharmacy, 300041 Timisoara, Romania; 4 Fondazione Banca Degli Occhi del Veneto, Via Paccagnella, 11, 30174 Venice, Italy; 5 Clinical Department of Pediatrics, “Carol Davila” University of Medicine and Pharmacy, Strada Dionisie Lupu No. 37, 020021 București, Romania

**Keywords:** age-related macular degeneration, fundus autofluorescence, optical coherence tomography, retinal pigment epithelium, scanning laser ophthalmoscope, multimodal imaging

## Abstract

**Background:** Age-related macular degeneration (AMD) is one of the leading causes of permanent vision loss in the elderly, particularly in higher-income countries. Fundus autofluorescence (FAF) imaging is a widely used, non-invasive technique that complements structural imaging in the assessment of retinal pigment epithelium (RPE) integrity. While optical coherence tomography (OCT) remains the gold standard for retinal imaging due to its high-resolution cross-sectional visualization, FAF offers unique metabolic insights. Among the FAF modalities, blue light FAF (B-FAF) is more commonly employed, whereas green light FAF (G-FAF) provides subtly different image characteristics, particularly improved visualization and contrast in the central macula. Despite identical acquisition times and nearly indistinguishable workflows, G-FAF is notably underutilized in clinical practice. **Objectives:** This narrative review critically compares green and blue FAF in terms of their diagnostic utility relative to OCT, with a focus on lesion detectability, macular pigment interference, and clinical decision-making in retinal disorders. **Methods:** A comprehensive literature search was performed using the PubMed database for studies published prior to February 2025. The search utilized the keywords fundus autofluorescence and age-related macular degeneration. The primary focus was on short-wavelength FAF and its clinical utility in AMD, considering three aspects: diagnosis, follow-up, and prognosis. The OCT findings served as the reference standard for anatomical correlation and diagnostic accuracy. **Results:** Both FAF modalities correlated well with OCT in detecting RPE abnormalities. G-FAF demonstrated improved visibility of central lesions due to reduced masking by macular pigment and enhanced contrast in the macula. However, clinical preference remained skewed toward B-FAF, driven more by tradition and device default settings than by evidence-based superiority. G-FAF’s diagnostic potential remains underrecognized despite its comparable practicality and subtle imaging advantages specifically for AMD patients. AMD stages were accurately characterized, and relevant images were used to highlight the significance of G-FAF and B-FAF in the examination of AMD patients. **Conclusions:** While OCT remains the gold standard, FAF provides complementary information that can guide management strategy. Since G-FAF is functionally equivalent in acquisition, it offers slight advantages. Broader awareness and more frequent integration of G-FAF that could optimize multimodal imaging strategies, particularly in the intermediate stage, should be developed.

## 1. Introduction

Age-related macular degeneration (AMD) is a chronic, complex, and multifactorial disease that affects the macula, the central region of the retina, resulting in a gradual deterioration of visual acuity [1]. AMD constitutes a significant public health concern because of its profound effect on the quality of life and the increasing patient population.

AMD is one of the leading global causes of permanent blindness in people aged 50 years and older with 1.8 million cases in 2020 [2]. By 2040, the number of people living with AMD worldwide is expected to significantly increase to nearly 300 million cases [3].

The current preferred method for the diagnosis and follow-up of individuals with AMD is using multimodal assessment. The gold standard is optical coherence tomography (OCT), which can be either spectral domain (SD) or swept source (SS). Additional investigations have encompassed the following: fundus autofluorescence (FAF), retromode imaging (RMI), OCT angiography (OCT-A), microperimetry, and color fundus photography (CFP). For selected cases of patients with neovascular AMD, imaging can include more invasive methods like fluorescein angiography (FA) or indocyanine green angiography (ICGA).

With regard to FAF, to our knowledge, the present guidelines for early and intermediate AMD lack consensus on its application. While recent documents such as the 2024 AAO Preferred Practice Pattern [4] acknowledge FAF as a useful imaging modality, particularly for detecting RPE abnormalities and monitoring geographic atrophy (GA) progression, they fall short of offering clear guidance on its practical implementation including aspects such as wavelength selection or standardized imaging protocols. Although FAF plays an established role in clinical trials, especially in the evaluation of GA, its integration into routine clinical pathways remains limited and non-uniform, even in the context of neovascular AMD. To date, no official guidelines more recent than the 2014 consensus authored by Schmidt-Erfurth et al. [5] have addressed the role of FAF in the management of neovascular AMD. This document briefly acknowledged FAF as a non-invasive imaging modality capable of revealing retinal pigment epithelium (RPE) alterations, but did not recommend its routine use in clinical practice nor provide technical details such as wavelength choice. In the absence of more recent formal recommendations, the most up-to-date insights on FAF application in AMD stem from expert opinion such as the presentation by Dr. Vujosevic at the 2024 EURETINA Congress [6]. There, FAF was discussed as a valuable tool for the diagnosis and monitoring of dry AMD, with particular emphasis on its technical limitations in imaging the foveal area and the absence of any specific recommendation regarding the use of blue versus green excitation wavelength. We wish to underscore the significance of this imaging method in real-world usage. The results of this analysis may function as a guide for ophthalmologists, facilitating the integration of short wavelength (SW) FAF into daily clinical practice for AMD patient management. The disease burden is substantial, the time allocated for each patient is constrained, and the resources are also limited. It is important to consider that FAF is rapid and non-invasive, although the discomfort may continue to be unpleasant for elderly patients. Given the availability of blue light (B-FAF) and green light FAF (G-FAF) with state-of-the-art devices nowadays, practitioners must decide which modality to use for various pathologies. Given the impracticality of employing both imaging types for every patient diagnosed with AMD and the lack of unanimity on the issue, a review of this nature would be valuable. This research sought to clarify the differences between two comparable varieties of FAF and emphasize their complimentary function to SD/SS-OCT in clinical practice.

## 2. Materials and Methods

We conducted a comprehensive review of the literature by searching the PubMed database of the National Institutes of Health using the following combination of keywords: fundus autofluorescence and age-related macular degeneration on 11 November 2024. We applied filters for English language, human studies, and available abstracts.

**Figure 1:**
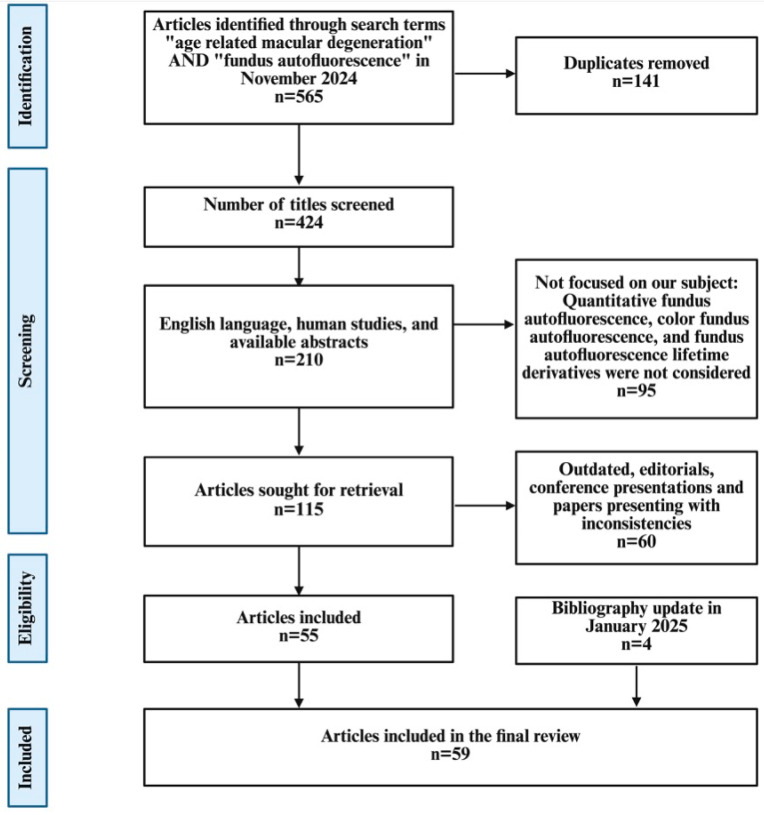
Flow diagram showing the article selection process. Note: The literature search covered studies published between 1995 and 2025 to capture both the early development and evolution of FAF imaging in AMD.

After reviewing the titles and abstracts, we selected studies that focused on fundus autofluorescence in AMD and imaging techniques as well as those exploring the pathophysiologic, diagnostic, and monitoring aspects of the disease. The studies involving quantitative FAF, color FAF, and FAF lifetime derivatives were not considered (Figure 1).

The PRISMA 2020 flow diagram templates are distributed in accordance with the terms of the Creative Commons Attribution (CC BY 4.0) license, which permits others to distribute, remix, adapt and build upon this work, for commercial use, provided the original work is properly cited. To view a copy of this license, visit https://creativecommons.org/licenses/by/4.0/ (accessed on 16 June 2025).

To establish correlations among various types of investigations and to comprehend the significance of merging FAF with OCT, we opted to include photos from our database of patients in different stages of AMD. We carefully selected photographs similar to those from the literature. The images were obtained using a scanning laser ophthalmoscope (SLO) Mirante (Nidek Co., Gamagori, Japan), which generates images with a field of view of 40 degrees. Three distinct laser wavelengths—blue (488 nm), green (532 nm), red (670 nm)—along with a specialized sensor for each of these wavelengths, were employed to produce color SLO images [7,8,9]. The Mirante platform was also utilized to obtain the SD-OCT images. The multimodal images were acquired in the clinic as part of the routine diagnostic and monitoring protocol for our patients with retinal disease. A highly skilled retinal specialist examined every image in line with the diagnostic criteria to find representative and similar cases that aligned with those that had already been documented in the literature. Patient written consent was obtained in accordance with the Declaration of Helsinki and approved by the ethics committee of our hospital.

## 3. Results

### 3.1. Age-Related Macular Degeneration Diagnosis

The clinical diagnosis of AMD generally entails an examination using a lens that directs light from a slit lamp through the pupil after dilation, known as biomicroscopy of the ocular fundus [10].

CFP has long been a conventional imaging technique for patients with AMD [11] and serves as an essential tool for documentation in both clinical trials and standard clinical settings, enabling effective comparisons during follow-up appointments [10]. CFP was replaced in the last decade by color images acquired by a confocal SLO, which is less affected by media opacities and can be executed with undilated pupils [12].

Over the last twenty years, OCT and FAF have been employed to identify retinal lesions, reaching an increasingly better resolution [1,13]. SD-OCT is a non-invasive diagnostic technique that provides in vivo, cross-sectional, high-resolution images by employing infrared light with a wavelength of 800–840 nm [14,15,16]. SD-OCT is used to assess particular morphological changes of the retina and subretinal areas that are important for both the progression of the disease and visual acuity [14,15,16].

FAF is a non-invasive imaging modality that has the capacity to illustrate the density map of normally and pathologically occurring fluorophores in the posterior pole [13,17,18]. In recent years, FAF has become a very important tool in the diagnostic and monitoring armamentarium for retinal diseases such as AMD [19,20,21]. Commercially available FAF systems are present on fundus cameras and confocal SLOs.

FA is an effective technique for identifying choroidal neovascularization (CNV) as well as its location and activity [1]. However, in recent years, FA has mostly been replaced by retina specialists with OCT-A [22]. OCT-A is a relatively new technique that can detect retinal and CNV and possesses the benefit over FA of being non-invasive and requiring no dye [23]. Another relatively new technology for AMD diagnosis and monitoring is retromode imaging, which can be acquired with an SLO that employs a laser light with an offset aperture instead of a central aperture, resulting in a pseudo-three-dimensional image [7,8].

The utilization of multimodal imaging offers further insights into retinal lesions, such as the identification of anatomical characteristics that enhance our comprehension of the disease mechanisms associated with the progression to late AMD [24,25].

### 3.2. Fundus Autofluorescence

Autofluorescence refers to the intrinsic capacity of certain endogenous biological molecules to emit light in the ultraviolet, visible, or near-infrared spectrum after absorbing excitation light at shorter wavelengths [26]. This naturally occurring phenomenon does not require external markers and is widely used in biomedical imaging, with each fluorophore characterized by a specific excitation and emission spectrum [26]. FAF is a quick, non-invasive method that reveals the fluorophores in the posterior pole. The main fluorophores found in the retina are lipofuscin, melanin, and melanolipofuscin [27]. The use of FAF in the detection and tracking of retinal diseases, particularly AMD, has become more and more significant since Delori’s initial description in 1995 [18]. Upon excitation, typically with blue light, fluorophores, like lipofuscin in the RPE, absorb photons, resulting in electron excitation. Upon returning to the ground state, electrons emit light at a longer wavelength [13]. FAF captures the emitted light to generate a spatial representation of fluorophore distribution [13].

**Figure 2:**
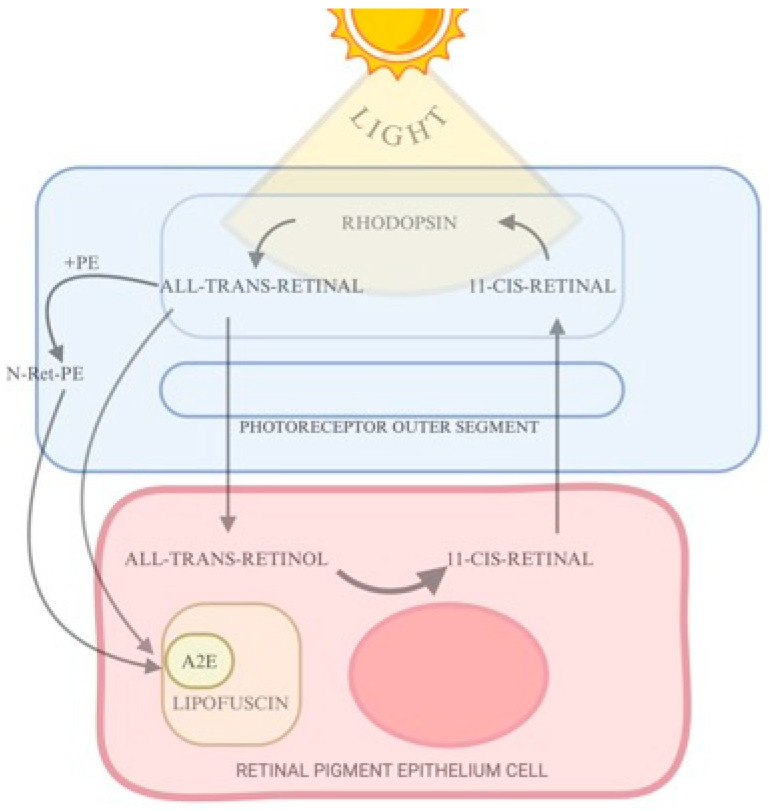
Simplified retinoid cycle. When exposed to light, 11-cis-retinal inside the photoreceptor attached to rhodopsin transforms into all-trans-retinal. Most of the all-trans-retinal is converted to all-trans-retinol and delivered to the RPE, where it is transformed again into 11-cis-retinal, which is returned to the outer segment of the photoreceptor. Excess all-trans-retinal combines with PE, and forms N-Ret-PE. All-trans-retinal binds with N-Ret-PE to form an intermediate compound, and it will be phagocytosed by the RPE. Inside the RPE, A2E, which is derived from the second compound, will accumulate and induce damage to the RPE. PE = phosphatidylethanolamine; N-Ret-PE = N-retinylidene-PE; A2E = N-retinyl-N-retinylidene ethanolamine, a lipofuscin component. Created in BioRender [29].

The RPE comprises a monolayer of polygonal cells that lies above the choroid and under the neurosensory retina. The RPE is important in retinal health maintenance, with lysosomal activity being essential to its function. Each day, RPE cells perform the phagocytosis of shed photoreceptor outer segments. The number of outer segments that each RPE cell phagocytoses throughout a lifespan is three million [28]. The retinoid cycle, also known as the visual cycle, is a thorough process; however, there are intermediate by-products that require further breakdown. Nonetheless, a minor fraction of these by-products is chemically incompatible for degradation and will consequently build up in the lysosomes of the RPE. Lipofuscin is the name of this undigested fraction [27]. Lipofuscin is the principal substance visualized using SW autofluorescence. Bisretinoid compounds, which are produced by the metabolism of vitamin A and the visual cycle, are the source of lipofuscin’s autofluorescent properties [13]. Bisretinoids are initially synthesized in the outer segments of photoreceptors and are subsequently deposited in the RPE as lipofuscin [18] (Figure 2 [29]).

**Figure 3:**
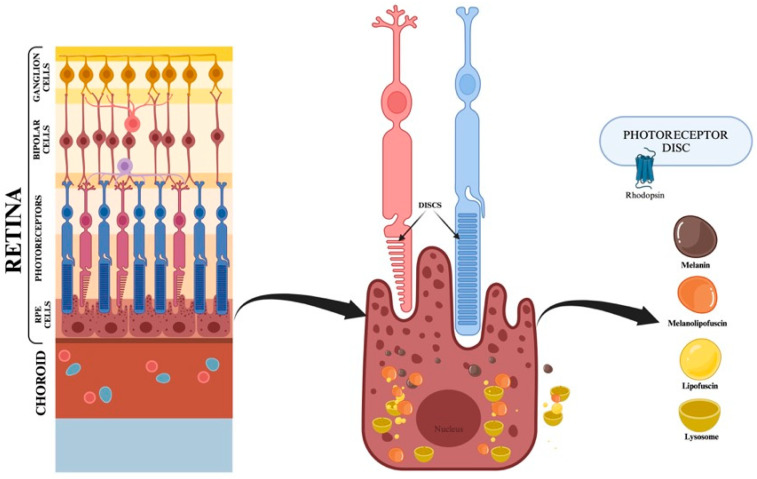
Granules of melanin, lipofuscin, and melanolipofuscin inside the RPE cells. Created in BioRender [31].

Granules of lipofuscin and melanolipofuscin, the conjugated product of lipofuscin and melanin, may occupy up to 20–33% of the free cytoplasmic space of the RPE cells over the age of 70 [30] (Figure 3 [31]).

Melanin is an ophthalmic pigment that is present within RPE cells and uveal melanocytes. The concentration of choroidal melanin increases from the periphery to the posterior pole. The concentration of RPE melanin diminishes from the periphery to the posterior pole and is also increased at the macular level [32].

Rhodopsin is a visual pigment that is concentrated in the outer segments of rod photoreceptors [13]. A brief removal of rhodopsin’s barrier to autofluorescent fluorochromes in the RPE can lead to autofluorescence augmentation, principally resulting from the repeated excitation by 532 nm light, which is called the bleaching effect [27].

The distribution of autofluorescence is a direct result of the distribution of lipofuscin and is most pronounced in the posterior pole. Autofluorescence is reduced in the fovea and also decreases as it approaches the periphery [13,18]. The accumulation of lipofuscin granules in human RPE is progressive with aging [33]. In degenerative retinal disorders, especially AMD and in macular dystrophies such as Best and Stargardt disease, the lipofuscin levels increase [13,33]. The first and most well-characterized substance of lipofuscin is N-retinyl-N-retinylidene ethanolamine (A2E), which has a negative effect on RPE cells [34]. A2E exhibits maximal emission at 560–575 nm and excitation at 430–450 nm [13,35]. A2E undergoes photo-oxidation when exposed to blue light, producing reactive oxygen species that can compromise cellular membranes, disrupt cholesterol metabolism, and induce apoptosis [36]. A2E is enzymatically indigestible, builds up in RPE lysosomes, and has been correlated with numerous degenerative retinal diseases [13].

FAF is an imaging technique that offers additional information compared with other retinal imaging techniques [37]. This technology can provide the clinician with unique insights about the retina, with FAF images reflecting the RPE and photoreceptors’ health as a topographic distribution of lipofuscin, the primary fundus fluorophore located in the RPE lysosomes [18]. Lipofuscin exhibits peak excitation at 470 nm and emits at a peak wavelength of 600–610 nm, which is yellow-green light [13,38]. Lipofuscin is situated basolaterally, while melanin granules are situated apically within the RPE cells. Melanin is the most prominent fluorophore in near-infrared (NIR) autofluorescence and has a maximal excitation at a longer wavelength of 787 nm [13]. Macular pigments, lutein and zeaxanthin, serve as antioxidants and filter blue light, with an absorption spectrum ranging from 400 to 540 nm and a peak at 460 nm, aimed at protecting the retina [13].

In summary, NIR-FAF and SW-FAF provide complementary insights about retinal health. While NIR-FAF is the best for detecting the melanin pigment distribution, SW-FAF is an adequate method for mapping the lipofuscin pigment in the RPE.

The American Academy of Ophthalmology has long advised of the incorporation of FAF into imaging protocols as a component of multimodal imaging for patients with late AMD [21], and it has proven to be helpful for evaluating the starting point of treatment and estimating the condition’s severity [39,40,41,42]. FAF is useful for assessing other retinal diseases such as central serous chorioretinopathy (CSCR) [43], macular dystrophies [44,45,46,47], cone-rod dystrophies [48], retinitis pigmentosa [49,50], macular telangiectasia type 2 [51], white dot syndromes [52], choroideremia [53], choroidal melanocytic lesions [54], and chloroquine/hydroxychloroquine retinopathy [55].

**Figure 4:**
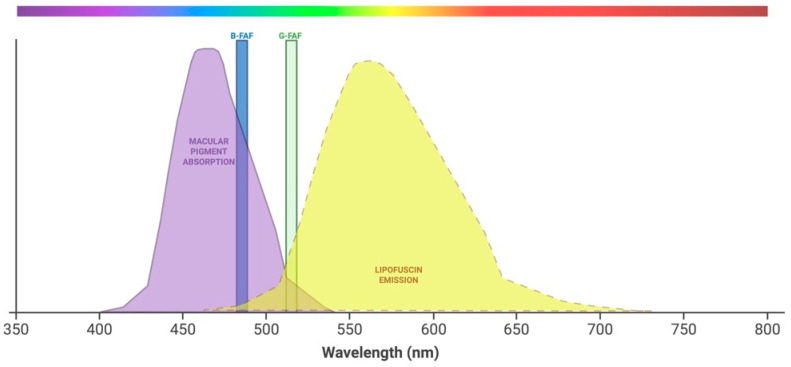
Spectral characteristics of B-FAF, G-FAF, and retinal fluorophores. Created in BioRender [56].

To better understand the spectral specificity of FAF imaging, Figure 4 illustrates the excitation and emission profiles of important retinal fluorophores and the wavelengths employed in B-FAF and G-FAF. B-FAF utilizes an excitation wavelength of 488 nm, with emitted light captured above 500 nm, primarily in the green spectrum. In contrast, G-FAF operates with an excitation at 514–532 nm with maximum emission detection around 633 nm, targeting longer wavelengths in the red spectrum.

#### 3.2.1. Fundus Autofluorescence in Normal Eyes

The outcome of FAF is an “en-face” image that illustrates a map depicting the arrangement and intensity of the autofluorescence of the retinal fluorophores across the fundus (Figure 3).

In the normal fundus, diffuse autofluorescence is observed in adult patients, with the retinal blood vessels and optic disc appearing as dark shadows [17]. The retinal vessels display significantly diminished FAF signal due to the obstructive effect of blood on autofluorescence. The optic nerve exhibits hypoautofluorescence due to the lack of RPE and its retinoid-derived lipofuscin. The FAF signal is decreased at the foveal region due to absorption by luteal pigment [28].

**Figure 5:**
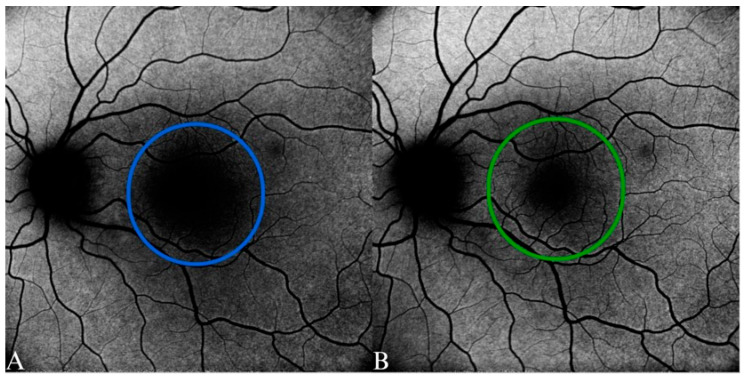
Fundus autofluorescence of normal fovea in a 38-year-old female patient. (**A**) Blue-FAF. (**B**) Green-FAF. The FAF signal was significantly reduced in A compared with B; the larger hypoautofluorescence area was due to higher absorption by the macular pigments.

The G-FAF pictures are optimal for the precise assessment of minor, central pathological alterations and for ascertaining the dimensions of a central big lesion [57]. Relying solely on B-FAF may result in an exaggerated assessment of the dimensions of atrophic patches and central involvement as it implies the existence of atrophy in the fovea [57] (Figure 5).

#### 3.2.2. Causes of Abnormal Fundus Autofluorescence

Depending on the underlying retinal pathology, lesions can be either hyperautofluorescent, hypoautofluorescent, or iso-autofluorescent [13,28,58].

Causes of hyperautofluorescence include:

- Increased lipofuscin inside the RPE;
  ○ Macular dystrophies: Stargardt dystrophy [47], pattern dystrophy [45];
- Subretinal autofluorescent deposits: vitelliform dystrophy [44];
- Window defect (pigment depletion): macular telangiectasia type 2 [51], white dot syndromes [59];
- Presence of other autofluorescent material: optic disc drusen [60], astrocytic hamartoma [61].

Hypoautofluorescence causes include:

- Luteal pigment, retinal vessels [28];
- RPE lipofuscin diminished or nonexistent: RPE atrophy (GA; CSCR) [43];
- Increased melanin inside RPE cells: congenital hypertrophy of RPE [62];
- Obstruction from material anterior to the RPE (cornea, anterior chamber, lens or the vitreous):
  ○ Macular pigments [28];
  ○ Fibrosis/scar tissue [28];
  ○ Media opacities [28];
  ○ Intraretinal/subretinal fluid [28];
  ○ Intraretinal/subretinal hemorrhage [28].

### 3.3. Fundus Autofluorescence in Age-Related Macular Degeneration

AMD is characterized by heterogeneous and pathognomonic signs, contingent upon the patient’s disease stage, and with various rates of progression over time. This paper sought to emphasize each clinical entity associated with AMD to evaluate the efficacy of FAF in diagnosing and monitoring patients as reported in the literature and provide suggestive multimodal imaging representations for the various clinical entities.

The same methodology should be used by every clinician to define the clinical manifestations and stages of AMD. In 2013, the Beckman Initiative for Macular Research Classification Committee released a consensus report delineating a clinical classification of AMD intended to offer universally comprehensible terminology for every ophthalmologist [63]. The Beckman classification (Table 1) necessitates either a clinical examination or a color fundus imaging to categorize AMD patients. Notwithstanding this endeavor, a deficiency in uniformity regarding the classification of AMD disease stages persists. A retina without drusen or pigment irregularities exhibits no signs of aging [63]. Small drusen are not classified as AMD, but rather as a typical aspect of macular aging [1,63].

The historical progression and pivotal contribution of key studies that helped establish FAF as one of the most important imaging modalities in the diagnosis and monitoring of patients with AMD are outlined below (Table 2).

#### 3.3.1. Early and Intermediate Age-Related Macular Degeneration

A study performed by Cocce et al. indicated that challenges in executing tasks in low light and low-contrast environments, including as dimly lit rooms and during dark adaptation, may work as indicators distinguishing early to intermediate stages of dry AMD [81], while certain patients may have slight visual distortion during nearby activities such as reading [1]. The presence of medium drusen is the primary clinical observation of early AMD. Patients diagnosed with intermediate AMD exhibit large drusen accompanied by visible pigmentary abnormalities in addition to medium drusen.

Numerous researchers have investigated FAF abnormalities in patients with early AMD [64,66,82,83,84,85]. Alterations in FAF were not consistently linked to funduscopically discernible drusen or pigmentation abnormalities, suggesting that FAF imaging could offer superior insights compared with other imaging modalities, being able to display more clear RPE modifications [66,86]. In patients with early AMD, topographic changes in FAF were categorized into eight distinct patterns by The International Fundus Autofluorescence Classification Group (IFAG) 20 years ago [66]. Apart from normal FAF, the images with alterations were classified as minimum change, focal increased, patchy, linear, lace-like, reticular, and speckled patterns [66] (Table 3). Minimal change, linear, lace-like, and speckled FAF patterns have a slow progression rate to late AMD [86].

Drusen are trademark lesions of AMD, which can be divided into five categories based on their size, consistency, and margin: hard drusen, soft drusen, calcified drusen, cuticular drusen, and pachydrusen. Another entity named SDD is also referred to as reticular pseudodrusen [87].

Hard drusen are distinguishable yellow-white deposits that are situated above Bruch’s membrane and behind the RPE. They possess a diameter of less than 63 μm, have clearly defined edges, and demonstrate a homogenous color density from the center to the periphery [88,89].

**Figure 6:**
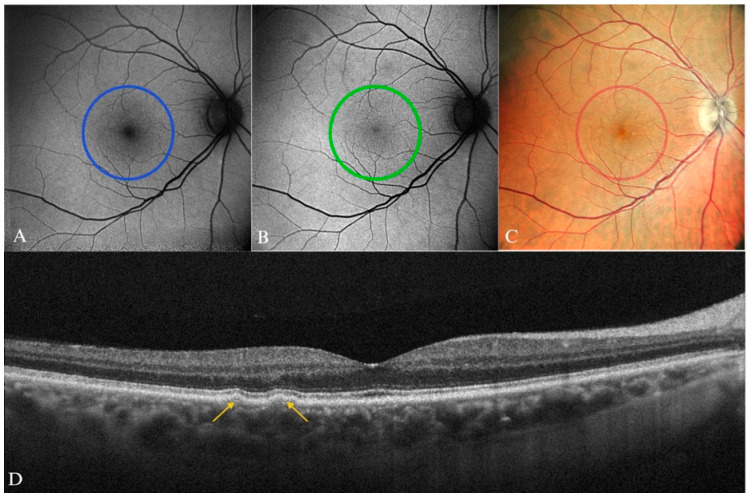
Early AMD in a 65-year-old male patient. (**A**) B-FAF and (**B**) G-FAF—with normal aspect in a patient with small- and medium-size drusen, which was barely visible on the (**C**) color SLO imaging (cSLO). (**D**) Yellow arrows indicating the lesions well-delineated on SD-OCT.

**Figure 7:**
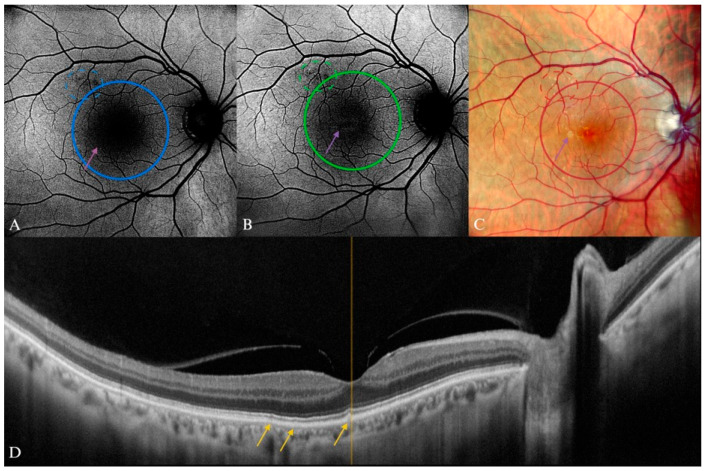
Early AMD in a 78-year-old male patient. (**A**) B-FAF and (**B**) G-FAF displaying “minimal change” pattern in the FAF imaging, which were more visible on (**B**) G-FAF, minimal alteration of the fundus autofluorescence; (**C**) purple arrow pointing to corresponding drusen on cSLO. (**D**) Yellow arrows showing the drusenoid lesions on SD-OCT. Apart from the visible drusen, vitreomacular adhesion syndrome is also present in this image.

The FAF imaging a normal or minimal change pattern is expected to correspond to a patient with small and medium drusen and no pigment abnormalities (Figure 6 and Figure 7). In one study, G-FAF proved to have a very high specificity and positive predictive value in the detection of these lesions, although B-FAF was not evaluated in this study for comparison [76].

**Figure 8:**
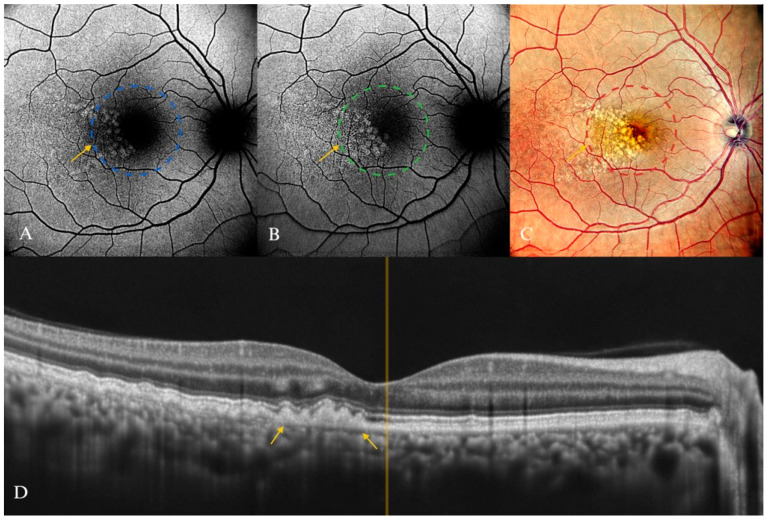
Intermediate AMD in a 74-year-old female patient. (**A**) B-FAF of multiple hyperautofluorescent round areas indicated by the yellow arrow, revealing a “patchy pattern” (inside the dashed circle). (**B**) In G-FAF, the luteal pigment had less impact, and thus the hyperautofluorescent round lesions were more clearly delineated, indicated by the yellow arrow (inside the dashed circle). (**C**) cSLO multiple soft drusen lesions (mostly visible inside the dashed circle). (**D**) SD-OCT displaying the corresponding aspect (yellow arrows).

Soft drusen (Figure 8) are less clearly defined compared with hard drusen, and they are typically characterized by round elevations with a diameter of 125 μm. The drusen’s central region may appear paler than its yellow edge [90]. The presence of soft drusen increases the probability of progression to end-stage atrophy, neovascularization, and loss of visual acuity [86,91].

Soft drusen appears to correlate to some degree with increased FAF areas [74,84,92]. Lois et al. suggested that drusen and AF might be independent indicators of aging in the macular region [92].

Cuticular drusen are composed of numerous small drusen that may form clusters. They were initially described in relation to their distinctive “starry sky” appearance during FA. They are strongly associated with AMD. These drusen are compact, hard, and of early onset. They have also been referred to as basal laminar drusen, although it has been claimed that the latter term is a misconception [87]. This type of drusen was characterized to be hyperautofluorescent with a hypoautofluorescent core in FAF imaging in a retrospective study [93].

It is probable that drusen formation is dependent on calcification, as the levels of calcium fluctuate over the course of their life cycle. The calcium content of drusen may increase as they ultimately regress, which is a consequence of the increasing chronicity [87]. It is believed that all drusen contain a small amount of calcium, which may be necessary for their formation. Consequently, calcified drusen may not be a distinct variety of drusen; more importantly, calcification may be associated with the age of the drusen [87]. Calcified drusen are described as white, clearly defined deposits on CFP, and on OCT, their distinctive feature is a hyporeflective core [94]. These lesions are linked to an elevated risk for the progression of AMD to GA [95]. On FAF, these lesions are mostly hypoautofluorescent [96].

Pachydrusen is a more recently recognized entity that was initially described by Spaide et al. in 2018 [97]. Located underneath the RPE, pachydrusen are depicted as solitary or dispersed yellow-white deposits with a regular outer contour [98]. OCT has confirmed that pachydrusen are an entirely novel entity that is distinct from soft drusen, as they have been reported to be associated with a thick choroid [97,98]. Pachydrusen has been demonstrated to have a predictive function in the progression of the disease in subsequent publications [99], indicating that they are a precursor to PCV, a hemorrhagic and exudative maculopathy that is a variant of neovascular AMD [100].

Drusen visualized using FAF can exhibit variable aspects and are mainly influenced by their size and the condition of the underlying photoreceptors [72]. Additional criteria deemed significant include drusen homogeneity, shape, reflectivity, and the presence of overlaying foci [72]. In a study conducted by Lois et al. [92], researchers identified an absence of a clear correlation between the distribution of drusen and FAF.

Spontaneous drusen regression is a marker of disease progression on SD-OCT, and it has been postulated that it precedes RPE atrophy. Retinal areas with such lesions are more likely to present with modified FAF patterns, hypoautofluorescence being more frequent than the increasing of FAF, as shown in a study using G-FAF [73]. Drusen associated atrophy is also related to reduced autofluorescence [101].

**Figure 9:**
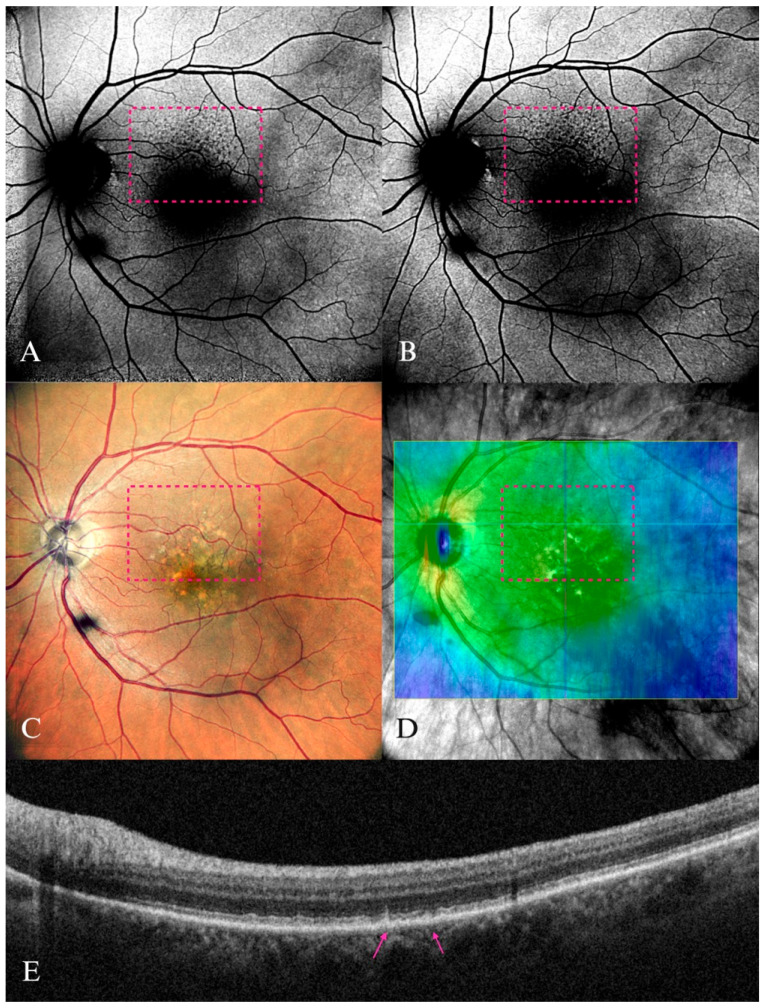
Subretinal drusenoid deposits in an 82-year-old female patient. (**A**) B-FAF and (**B**) G-FAF displaying the “reticular” pattern in the superior macula (in the pink dotted rectangle) as multiple small hypoautofluorescent dots. (**C**) Multiple drusenoid yellow lesions inside the pink dotted rectangle on cSLO. (**D**) En-face image displaying the location (the blue line inside the pink dotted rectangle) of the section in (**E**). Pink arrows point toward the SDD on SD-OCT.

SDD are pathological entities, which in contrast with drusen are located above the RPE. The terms “reticular drusen” [102] or “reticular pseudodrusen”, referred to as SDDs, have been employed in numerous cohort studies because it has been recognized as a significant risk factor for the progression of AMD to its advanced stages [103]. There are three distinct types of SDDs: dot, ribbon, and peripheral. SD-OCT is one of the best methods used in order to diagnose SDDs [104]. Despite the considerable effort required, the volume scan must be evaluated and each individual line scan inspected to accurately detect their presence in OCT, which is exceedingly time-consuming [21]. SDDs are associated with the “reticular pattern” on FAF imaging [61,62,65], and it is a highly sensitive marker for the risk of progression to CNV [66,105] (Figure 9). A study involving 100 eyes showed that G-FAF is an effective tool for describing SDDs. However, the B-FAF technique was not evaluated in this study [76].

Pigmentary abnormalities represent a significant characteristic of non-neovascular AMD and indicate an increased likelihood of development to atrophy and neovascularization [25].

**Figure 10:**
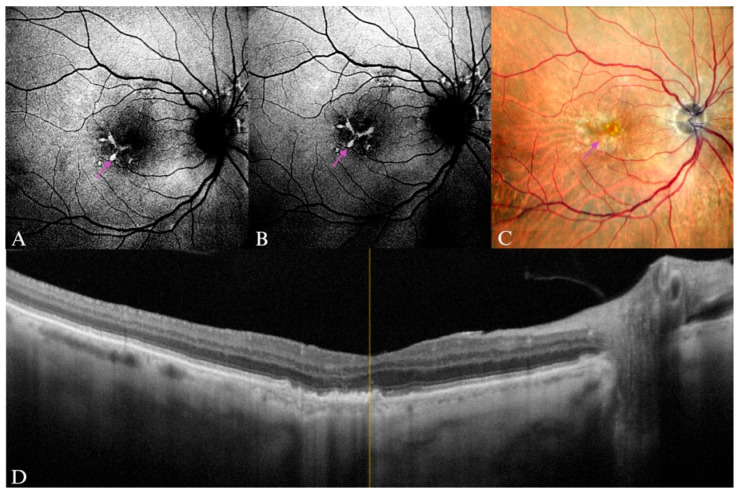
Pigmentary abnormalities in an 84-year old male patient, which reflect in (**A**) B-FAF, where the purple arrow points toward three visible lines forming a “linear pattern” of intense hyperautofluorescence. (**B**) In G-FAF, the hyperautofluorescent lesion line that passes through the foveal region is significantly more intense, with clear delimitation. (**C**) Drusenoid lesions, where the purple arrow pointing to the pigmentary abnormalities that seem to correlate with the hyperautofluorescent lesions in images (**A**,**B**). (**D**) On the SD-OCT image, irregular RPE and drusenoid lesions can be seen.

CFP has always been the best approach for assessing RPE abnormalities; however, FAF has recently been the favored modality for evaluating RPE atrophy [39]. Spaide employed a green-yellow FAF using a fundus camera with an excitation wavelength of 580 nm [64], demonstrating that this technique reveals the hyperautofluorescence caused by lipofuscin accumulation in individual or overlapping cells in the areas of hyperpigmentation seen on CFP [64] (Figure 10). Atrophic regions devoid of RPE exhibit hypoautofluorescence on FAF images [21]. SD-OCT can additionally be employed to assess and quantify pigmentary alterations [15,106].

Focal regions of RPE loss or depigmentation may be indicated by choroidal hypertransmission, and pigment clumping and movement may be observed as outer retinal hyperreflective foci on OCT [107].

**Figure 11:**
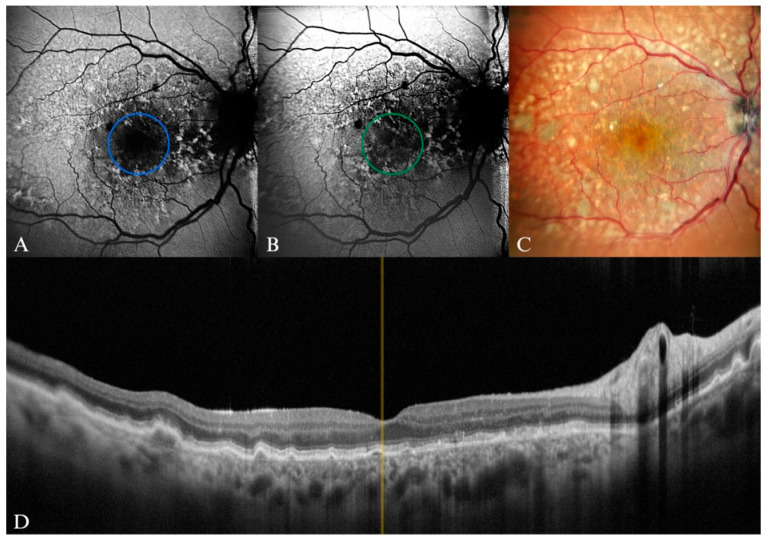
Intermediate AMD in a 77-year-old male patient. (**A**) B-FAF multiple hyper- and hypoautofluorescent lesions that spread beyond the temporal vascular arcades, however the central macular region is obscured in shadow (blue circle). (**B**) G-FAF of the same eye with an enhanced central area (green circle), displaying more clearly the same foveal lesions, arranged in a “speckled pattern”. (**C**) Soft drusen and pigmentary abnormalities spread from the foveal area and beyond the macula in cSLO imaging. (**D**) Multiple drusenoid lesions in a central scan through the fovea on SD-OCT.

A study conducted by the members of the Fundus Autofluorescence in Age-related Maculopathy Study Group (FAM Study Group) [86], who used the IFAG classification, provided supplementary information regarding FAF imaging interpretation and its correlation with funduscopic retinal features as well as its association with visual acuity and the prediction of disease development to a certain extent. They concluded that hyperpigmentation correlates with a FAF lacelike pattern and linear pattern [86]. Numerous pigmentary abnormalities were determined in patients with speckled pattern FAF (Figure 11).

The patchy pattern on FAF was mostly observed in patients with hyperpigmentation and drusen. The focal increased pattern FAF was noticed to be related to the presence of hyperpigmentation and soft drusen [86].

Midena et al. affirmed the association between intricate FAF patterns, soft drusen, and pigment irregularities, which consistently correlated with diminished retinal sensitivity on microperimetry, a functional assessment of the retina [108]. Querques et al. corroborated the association between decreased auto-fluorescence signal, photoreceptor damage indicated on SD-OCT, and reduced retinal sensitivity [109].

#### 3.3.2. Age-Related Macular Degeneration Prediction of Progression to the Late Stages

Progression of early and intermediate AMD to the late atrophic stage was associated with a focal and focal-plaque-like FAF pattern in patients who clinically had both drusen and hyperpigmentation [86].

Numerous researchers have investigated eyes with non-exudative AMD, documenting various FAF patterns as predictive variables for conversion to the exudative form of the illness, and also described FAF patterns in neovascular AMD (nAMD) [83,86,110]. Studies that monitored early and intermediate AMD patients determined that the most significant progression to the late exudative form of AMD was in patients with a baseline patchy, reticular, linear, and focal-plaque-like FAF pattern [86,110].

FAF is also valuable as a tool for classifying atrophic zones according to the rate of progression [111,112].

#### 3.3.3. Late Age-Related Macular Degeneration

The two variants of advanced disease are neovascular AMD (wet or exudative) and atrophic AMD (dry or non-exudative), although these can coexist. The characteristic lesions are macular neovascularization and GA, respectively. At this point in time, visual symptoms are frequently dramatic and irreversible, potentially resulting in considerably reduced central visual acuity in the affected eyes [113]. For most of the patients, a central scotoma is the final outcome from either the neovascular or non-exudative form of the disease.

##### Geographic Atrophy

GA arising from AMD is currently characterized by distinctly outlined atrophic lesion in the outer retina, caused by the degeneration of photoreceptors, RPE, and the underlying choriocapillaris, culminating in permanent vision impairment [114]. The GA area occupies at least a round 175 μm diameter in the macular region [89], with a progression rate of about 2 mm^2^/year [115,116,117]. Eyes with greater initial atrophy patches have consistently demonstrated higher rates of enlargement [115].

**Figure 12:**
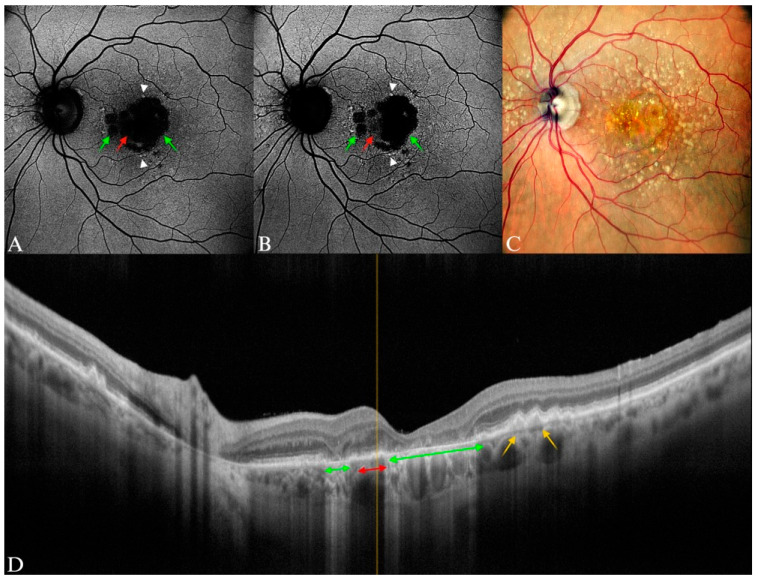
Late AMD, GA in an 82-year-old female patient. (**A**) Central hypoautofluorescent area of GA on B-FAF (between the green arrows), including the spared of atrophy area (red arrow). (**B**) Multiple round patches (green arrows), but with a partial foveal sparing obvious by comparison with B-FAF (red arrow). White arrow heads in (**A**,**B**) point to focal points of hyperautofluorescence. (**C**) Central GA, with visible choroidal vessels and hard and soft drusen areas extending beyond the macular region. (**D**) In the SD-OCT image, the green arrows correspond to the area of GA, with outer retinal atrophy and RPE atrophy and obvious hypertransmission. The red arrow corresponds to the spared nasal quadrant of the fovea, and the yellow arrows point toward to the soft, confluent drusen.

GA can be observed through various imaging modalities, displaying distinct features in each, such as greater apparent visibility of underlying choroidal vessels with a well-defined border on CFP or color SLO, hypoautofluorescence on FAF (Figure 12), with a subsidence of the outer retinal layers, RPE atrophy, and with increased choroidal transmission on OCT [114]. A pseudo three-dimensional round patch exhibiting uniform reflectivity, with distinctly visible hyperreflective choroidal vessels beneath the retina, is depicted in the retromode image [8,118].

FAF evaluation of GA progression serves as a reproducible [119] and dependable assessment instrument [112,120,121]. G-FAF provided superior inter-reader agreement than B-FAF in one study for assessing the lesion size due to enhanced visualization of the foveal area and less interference from the macular pigment [122]. FAF serves as an endpoint in clinical studies for novel medicines that currently provide hope for patients with the end-stage atrophic form of AMD [123,124]. The FAM Study Group conducted research on 149 eyes with GA [67]. As previously indicated, the GA region exhibited hypoautofluorescence on FAF imaging. Two-thirds of the examined eyes had a unifocal patch of GA, whereas one-third displayed multifocal regions of GA. Nearly fifty percent of the studied eyes exhibited GA affecting the fovea [67]. B-FAF is superior for peripheral GA lesions, but G-FAF provides a more accurate representation of central atrophy [57]. B-FAF more often overestimates the GA size [57,125], with the foveal shadowing being reduced in G-FAF imaging [118].

**Figure 13:**
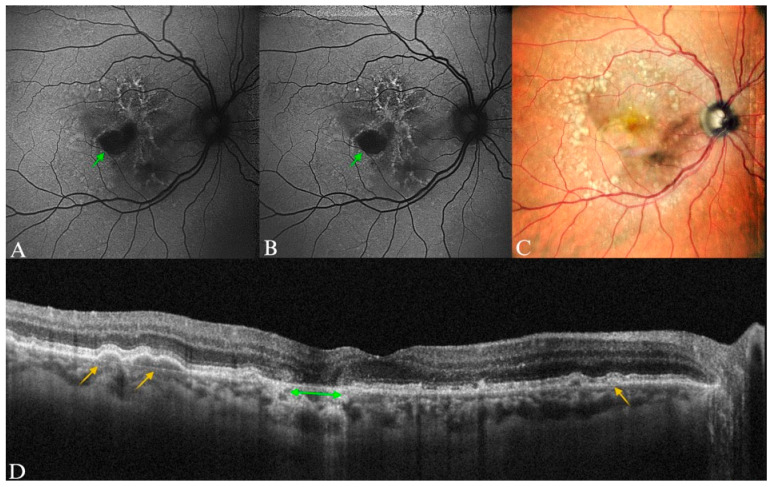
GA in a 67-year-old female patient. (**A**) “Branching” diffuse pattern of GA with a hypoautofluorescent area (green arrow) and adjacent branch-like hyperautofluorescent lesions on B-FAF. Upon initial observation, the fovea may seem affected by the GA region. (**B**) In the G-FAF image, the foveal sparing (green arrow) was more precise than in the first image. (**C**) Small area of GA, with visible choroidal vessels and multiple medium and large drusen. (**D**) Green arrow corresponds to the atrophic area, yellow arrow points to multiple drusenoid lesions.

Patients exhibiting an aberrant FAF pattern surrounding the GA region, referred to as the junctional zone, were categorized into many patterns: none, focal (Figure 10), banded, patchy, and diffuse. Initially, the diffuse pattern was categorized into four types: reticular, branching (Figure 13), fine granular, and fine granular with peripheral punctate spots [67], and a fifth named the diffuse trickling pattern, which was later described by Holz et al. [19] (Table 4).

The banded and diffuse patterns were defined as fast progressors, while focal pattern FAF was associated with a slower disease progression [67]. The diffuse trickling pattern demonstrated considerably faster progression than the other subtypes [19,126]. Although both procedures yielded comparable readings, G-FAF is often the superior option to B-FAF for evaluating patients with GA when a choice is available [57,122,125,127]. A limited investigation by Pfau et al. including 40 eyes showed that G-FAF was a marginally superior approach to B-FAF for the accurate characterization and quantification of GA [122].

SD-OCT measurements of the GA area proved to have a considerable association with the atrophy lesions quantified with FAF in a recent and reliable study conducted by Ehlers et al. [80]. The association of FAF and SD-OCT in patients with AMD is necessary for a good understanding of the disease process and a more accurate prognostic [71]. Bui et al. reinforced the fact that the association of these two investigations aimed at identifying relevant information regarding disease status using biomarker identification [77]. They concluded that SDDs, in conjunction with hyperreflective foci (HRF) on SD-OCT and particular FAF patterns, notably diffuse-trickling, serve as predictive indicators of GA growth [77]. Egger et al. [128] followed-up patients with GA using FAF and SD-OCT. A robust correlation was identified between diffuse and diffuse-trickling FAF patterns and the areas with absent RPE and photoreceptors, characterized by the rapid development rate of end-stage atrophic AMD [128].

Curcio et al. [117] eloquently elucidated the rationale underlying the FAF aspect concerning photoreceptor disease in a patient with drusen and GA. Photoreceptors that lost their original shape were correlated with an increased FAF signal, whereas atrophy and diminished FAF signal were observed in regions of severely degraded photoreceptors [117]. Their study results strongly supported the use of SD-OCT in conjunction with FAF.

##### Neovascular Age-Related Macular Degeneration

Exudative AMD encompasses various typical lesions, including intraretinal fluid, subretinal fluid, sub-RPE fluid, submacular hemorrhages, irregular RPE detachment, and the end-stage lesions, represented by disciform fibrotic scars [8]. nAMD is accountable for the majority of severe vision loss instances, despite the lower prevalence compared with atrophic AMD [2]. Type 1 or occult neovascularization occurs when choroidal neovascularization proliferation originates below the RPE and leaves a weakly defined leaking pattern on FA [1], and a more irregular autofluorescence pattern on FAF imaging [68,69]. Polypoidal choroidal vasculopathy (PCV), a distinct type 1 choroidal neovascularization subclassification, has a significant aneurysmal component [1]. Classic choroidal neovascularization exhibits intense fluorescein leakage, is type 2 neovascularization, it occurs above the RPE in the subretinal space, and presents with reduced FAF [68,69]. An anastomosis that connects the choroidal and retinal circulations causes type 3 neovascularization or retinal angiomatous proliferation (RAP) [1].

In patients with nAMD, unlike GA, the hypoautofluorescent region does not always correlate effectively with the scotoma. For incipient CNV lesions, the FAF might appear normal [68,70], which could reflect a good function [129] and health of the RPE and photoreceptors, which has a positive effect in what concerns the visual outcome [130]. Eyes with nAMD could also exhibit a decrease in autofluorescence due to the presence of intraretinal or subretinal fluid or blood, which exerts a blockage effect [69,70]. Another reason for decreased autofluorescence in nAMD is the atrophy of RPE [131] and photoreceptors [68]. Increased autofluorescence in exudative AMD can be attributed to photoreceptor depletion in areas affected by fluid and an augmented fundus autofluorescence signal from preserved RPE [132]. Research conducted on patients with exudative AMD indicated that following therapy with anti-vascular endothelial growth factor, an increase in autofluorescence can sometimes be observed [133]. Takasago et al. demonstrated that in individuals with treated nAMD, the atrophic region in the macula identified by FAF strongly coincided with the non-perfused choriocapillaris area assessed by OCT-A [75].

Disciform scars were described as mostly areas of reduced autofluorescence with hyperautofluorescent borders by Ruckmann et al. [83] and as significantly hyperautofluorescent areas by Kellner et al. [70]. Higher autofluorescence is more frequent in the fellow eye of patients with nAMD [64]. An interesting observation was made by Fujimura et al. [134], who noted that the congener eye of patients with nAMD tended to have an abnormal FAF pattern, while patients with PCV in one eye could present a relatively normal FAF pattern more frequently in the contralateral eye [134].

**Figure 14:**
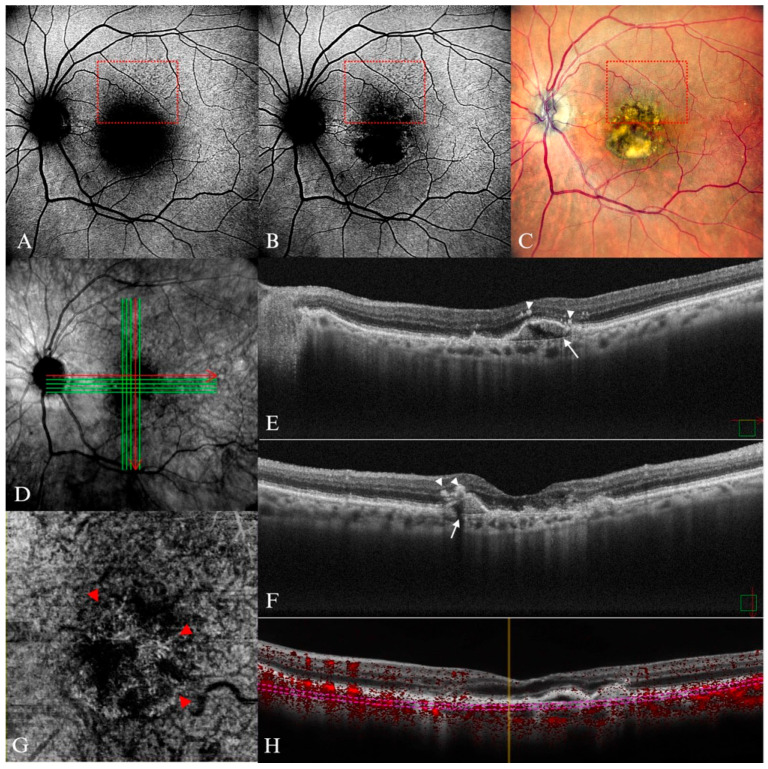
Neovascular AMD in an 82-year-old female. (**A**) Central reduced autofluorescence area and a visible reticular pattern on B-FAF in the upper quadrant delineated by the dotted red rectangle, (**B**) In G-FAF, the area of hypoautofluorescence is reduced and exhibits a clearer boundary. The dotted red rectangle contains the reticular pattern (**C**) SDD in the upper quadrant (dotted red rectangle) and central fibrovascular PED. (**D**) En-face SD-OCT image of (**E**,**F**) irregular mixed type PED (white arrow) with adjacent hyperreflective foci (arrow heads) (**E**) Represents the horizontal section (corresponds to the horizontal red arrow in (**D**)), while (**F**) represents the vertical section (and corresponds to the vertical red arrow in (**D**)). (**G**) Choroidal neovascularization visible in en-face OCT-A image, and (**H**) in section of OCT-A.

Pigment epithelium detachment (PED) is a frequent lesion found in patients with non-exudative or exudative AMD [135,136]. PED is defined as a separation of the retinal layers, between the RPE and Bruch’s membrane [78]. Multimodal imaging for the diagnosis, characterization, and classification of PED may encompass various non-invasive techniques like SD-OCT, FAF, and OCT-A [78]. Depending on their contents, PED can be serous, drusenoid, vascularized, hemorrhagic, and mixed [78]. Irregular autofluorescence, uniform hyperautofluorescence, normal, and hypoautofluorescence patterns have been described to correspond with the PED [78,137]. The FAF variable aspect is likely to be associated with the other modified retinal layers [78], which is why the multimodal approach is indicated (Figure 14).

RPE tear is a complication that may occur in eyes with large fibrovascular PED spontaneously or following anti-VEGF therapy, as the neovascular membrane undergoes contraction [138,139,140,141]. In OCT, the RPE tear can be seen as a region of RPE dehiscence alongside elevated PED characterized by a retracted and irregular RPE monolayer [140]. In FAF, the RPE tear manifests as a region of hypoautofluorescence in the region with bare choroid, where the RPE is entirely absent, accompanied by an adjacent zone of hyperautofluorescence where the RPE margin is torn and retracted [70,141].

Submacular hemorrhage is a rare, acute, and sight-threatening complication in the context of nAMD. The FAF signal in the area of concern is going to exhibit alterations, based on the period of time prior to receiving treatment. The FAF patterns can be either hypo- or hyperautofluorescence, caused by the RPE and photoreceptor damage [79].

The following table (Table 5) outlines the complementary roles of FAF and OCT in clinical practice, specifically in the detection and characterization of retinal lesions associated with AMD. OCT delivers comprehensive cross-sectional structural data, whereas FAF presents a functional and metabolic representation.

### 3.4. Blue Fundus Autofluorescence Versus Green Fundus Autofluorescence in Age-Related Macular Degeneration Patients

The key studies that have investigated the comparative performance of B-FAF and G-FAF in patients with AMD are summarized in the table below (Table 6).

Advances in ophthalmic imaging have led to the development of multimodal platforms that integrate complementary technologies into a single device. Systems combining OCT, FAF, confocal SLO, and FA/ICGA allow for a comprehensive evaluation of the retina. The table below (Table 7) highlights several widely used imaging platforms, emphasizing their key features and capabilities in clinical practice.

## 4. Discussion

The purpose of this review was to summarize the current information regarding the variations of one important retinal imaging technology used for AMD assessment. The capturing process and primary retinal pigments were briefly discussed, as comprehending these principles is essential for interpreting the final results and analyzing these images in practice.

We want to emphasize that FAF is an exceptional tool; although its application in real-world practice may be time-consuming and potentially stressful for patients, it is valuable due to its non-invasive nature and relatively swift execution, providing crucial and distinctive insights into the retinal health of patients with AMD.

FAF intensity in the central retina is reduced when the blue excitation wavelength is used due to the absorption carried out by the macular pigment. In contrast, the laser operating in the green spectrum has the advantage that there is less absorption by the macular pigment, less obstruction effect from the media opacities, and less discomfort reported by the patients as well as diminished corneal and lens autofluorescence. Considering the characteristics of AMD, the primary emphasis is on the status of the central retinal region, where every micron is significant and relevant for the patient and even for treatment decisions.

The observation that changes in FAF are not always connected with visible fundus abnormalities in patients with AMD underscores the uniqueness of this investigation and its essential role in the retinal clinician’s arsenal.

Currently, there is limited information about G-FAF usage in patients with early and intermediate AMD. Since the RPE and photoreceptors are more preserved at these stages, this imaging technique could facilitate the recognition of further insights and enhance our knowledge about potential aggressors.

FAF imaging is complementary to OCT. Developing a multimodal approach is already documented, especially when using a SLO/OCT platform, which has become more available than ever before. Consistency and reproducibility are essential when building a database of images and accurately classifying diseases and their stages to appropriately interpret FAF patterns.

Regarding future research directions, prospective studies using G-FAF in early and intermediate AMD could yield valuable insights into RPE function and photoreceptor integrity. We propose the implementation of standardized imaging protocols that combine G-FAF with OCT, applied to carefully selected patients with different AMD phenotypes. Comparative evaluation with the more commonly used B-FAF may help delineate their advantages, particularly in the foveal region, where macular pigment absorption limits B-FAF signal. G-FAF, in contrast, may allow for an improved visualization of foveal structures and a more accurate assessment of disease extent.

## 5. Conclusions

G-FAF is insufficiently explored, so the photographs were meticulously and intentionally chosen to illustrate various aspects of this prevalent blinding condition.

This article is the first to have presented G-FAF, B-FAF, color SLO, and SD-OCT images of every AMD stage to our knowledge, as most of the articles have clearly focused on GA.

This review paper aims to encourage every retina clinician to utilize G-FAF regularly, which, to our knowledge, could potentially lead to novel discoveries concerning the pathophysiological mechanisms of AMD.

Given the considerable burden of AMD, we expect that forthcoming trials will employ the G-FAF for individuals with intermediate AMD, who are at the highest risk of visual acuity loss while possessing the capacity to prevent it.

## Figures and Tables

**Table 1:** Beckman classification of AMD [1,63].

AMD Stage	Clinical Findings
Early AMD	Medium drusen, with dimensions >63 μm and ≤125 μm Absence of any pigment abnormalities [63]
Intermediate AMD	Large drusen, with >125 μm and/or the presence of pigment abnormalities [63]
Late AMD	GA and/or exudative AMD [63]

**Table 2:** Historical milestones regarding FAF usage in AMD.

Year	Study	FAF System, Excitation Wavelength	Milestone	Contribution
1995	Delori et al. [18]	Custom-built fundus spectrophotometer; 430–550 nm	In vivo characterization of FAF in human retina.	Created the biochemical and biophysical foundation for FAF imaging in retinal diseases. Postulated that lipofuscin is the main retinal fluorophore and established the localization and distribution of autofluorescence.
1995	Ruckmann et al. [17]	Confocal SLO; 488 nm	Confocal SLO was used for in vivo FAF imaging of the retina.	FAF images presented enhanced contrast, and less interference. Confirmed that the autofluorescence signal primarily originated from lipofuscin.
2003	Spaide RF [64]	Modified fundus camera; 580 nm	Fellow eyes of patients with exudative AMD showed higher autofluorescence, suggesting FAF has a role in risk stratification before exudation.	Linked focal hyper-autofluorescence to areas of hyperpigmentation, indicating lipofuscin accumulation and oxidative stress. Supported the concept that FAF reflects the RPE stress, which may precede neovascularization.
2004	Schmitz-Valckenberg et al. [65]	Confocal SLO; 488 nm	Correlation of FAF signal abnormalities with retinal sensitivity in the GA junctional zone.	Functional correlation of FAF phenotypes in late form of AMD. FAF imaging represents a structural tool and a marker for functional decline in disease progression.
2005	Bindewald et al. [66]	Confocal SLO; 488 nm	Phenotypic classification of FAF patterns in patients with early AMD.	Eight FAF phenotypes were described: normal, minimal change, focal increased, patchy, linear, lacelike, reticular, and speckled. Retinal alterations which are not visible on color fundus photography can be seen on FAF imaging.
2005	Bindewald et al. [67]	Confocal SLO; 488 nm	Phenotypic classification of FAF patterns in the junctional zone of GA in AMD.	Identified five FAF junctional patterns: none, focal, banded, patchy, and diffuse—with the latter subdivided into reticular, branching, fine granular, and fine granular with peripheral punctate spots. A reproducible imaging-based phenotypic classification was created.
2005	Dandekar et al. [68]	Confocal SLO; 488 nm	Characterization of the FAF patterns of CNV due to AMD.	Preserved FAF signifies viable RPE and an improved visual prognosis, but reduced FAF shows RPE and photoreceptor loss. FAF is an effective instrument for evaluating disease progression and guiding treatment strategies.
2007	Holz et al. [19]	Confocal SLO; 488 nm	FAF phenotypes can predict the rate of GA progression in AMD.	GA progression assessment in relation to FAF patterns. They demonstrated that specific FAF phenotypes in the junctional zone—particularly diffuse trickling and banded—are associated with significantly faster atrophy enlargement.
2007	McBain et al. [69]	Confocal SLO; 488 nm	FAF patterns in untreated classic and occult CNV in AMD using confocal SLO.	Classic CNV presents with reduced FAF, likely due to blockage rather than RPE destruction. Occult CNV patients present with variable FAF aspect, reflecting the heterogeneous pathology.
2010	Kellner et al. [70]	Confocal SLO; 488 nm (SW-FAF) and 787 nm (NIR-FAF)	Comparative study of SW-FAF and NIR-FAF in AMD, using a single imaging system.	SW-FAF and NIR-FAF visualize different aspects of RPE pathology—lipofuscin (SW) vs. melanin (NIR). Found that NIR-FAF changes often precede or exceed SW-FAF loss, especially in exudative AMD.
2011	Schmitz-Valckenberg et al. [71]	Confocal SLO; 488 nm	Quantitatively correlated FAF-defined atrophic areas with OCT layer disruptions across the outer retina and RPE in GA.	Validated that severely reduced FAF corresponds to irreversible RPE and photoreceptor damage. Supported FAF as a reliable, non-invasive tool to monitor GA progression and assess structural-functional correlation.
2012	Landa et al. [72]	Confocal SLO; 488 nm	Directly correlated OCT morphological characteristics of individual drusen with their FAF aspect, using precise image registration techniques.	Identified drusen size and ellipsoid zone disruption as key factors strongly associated with abnormal FAF. Provided biomarkers for AMD progression. Supported FAF as an early indicator of photoreceptor-RPE dysfunction before visible atrophy.
2013	Toy et al. [73]	Modified fundus camera; 550–600 nm	Demonstrated that spontaneous drusen regression in intermediate AMD is frequently associated with significant localized FAF signal changes, even when fundus photographs appear normal.	Found that drusen regression showed decreased FAF, suggesting early RPE damage. Showed FAF is more sensitive than CFP for detecting post-regression changes.
2016	Göbel et al. [74]	Confocal SLO; 488 nm	Correlated FAF signal intensity over drusen with specific microstructural OCT alterations, helping refine imaging biomarkers in early and intermediate AMD.	Showed that drusen with abnormal FAF aspect often had corresponding structural damage on OCT. Highlighted FAF variability across drusen, reflecting different stages of AMD progression.
2019	Takasago et al. [75]	Confocal SLO; 488 nm	Demonstrated a strong association between macular atrophy areas seen on FAF and choriocapillaris non-perfusion on OCT-A in treated exudative AMD.	Showed that choriocapillaris non-perfusion area is larger than macular atrophy in most cases. Suggested that choroidal ischemia plays a major role in the pathogenesis of atrophy after anti-VEGF. Advocated for the concomitant utilization of FAF and OCT-A in evaluating structural and vascular abnormalities in AMD.
2020	Cozzi et al. [76]	Confocal SLO; 532 nm	Comprehensive study comparing five advanced imaging modalities, including G-FAF and retromode, for identifying and classifying drusen and subretinal drusenoid deposits (SDD) in early/intermediate AMD.	G-FAF was highly effective for detecting dot SDDs. Highlighted G-FAF’s value in multimodal classification—particularly useful alongside OCT.
2022	Bui et al. [77]	Confocal SLO; 488 nm	Established one of the first large-scale models integrating FAF and deep learning-analyzed OCT biomarkers to predict GA progression in patients with AMD.	Found that FAF patterns, especially the diffuse-trickling phenotype, and the presence of SDD were significantly associated with faster GA growth. Showed that HRF concentration, a marker of RPE dysmorphia, interacted significantly with FAF phenotypes in predicting GA progression.
2023	Bindewald-Wittich et al. [78]	Confocal SLO; 488 nm	Large-scale, systematic study which classified FAF patterns of PED for diverse underlying pathologies. Patients with AMD represented 85% of the total cases included.	Showed irregular/granular FAF as the most common pattern overlying PED. Concurring pathologies found in patients with AMD: drusen, hemorrhages, GA, RPE tears.
2024	Nawrocka et al. [79]	Confocal SLO; 488 nm	The study demonstrated that FAF patterns were not influenced by the treatment type, but rather by the duration of the hemorrhage prior to intervention.	Established that FAF imaging can reflect the age and metabolic state of subretinal hemorrhage, making it a valuable indicator of photoreceptor and RPE damage in AMD. Highlighted FAF as a non-invasive modality for assessing chronicity of SMH and guiding clinical decisions regarding urgency and prognosis.
2024	Ehlers et al. [80]	Confocal SLO; 488 nm	This study demonstrated a strong and consistent correlation between B-FAF and OCT measurements of GA over an 18-month period, using data from the GATHER1 clinical trial.	Validated OCT as a reliable alternative to FAF for quantifying GA area in clinical trials. Showed minimal differences between FAF and OCT-based GA measurements. Highlighted complementary strengths of OCT and FAF.

**Table 3:** Overview of FAF patterns in early and intermediate AMD, according to IFAG [66], with frequency and risk assessment [86].

FAF Pattern	FAF Description	Fundus Correlation [66]	Clinical Significance	Frequency
None/Normal [66]	No visible FAF changes	Hard/soft drusen	Low risk of progression	N/A
Minimum change [66]	Limited FAF intensity alterations	Hard/soft drusen	Slow progression	9%
Focal increased [66]	Punctiform, isolated area of hyperautofluorescence +/− dark halo	Pigment abnormalities/soft drusen	Documented cases progressed to GA	4%
Patchy [66]	Larger, irregular areas of hyper-FAF	Pigment abnormalities/soft drusen	High risk of progression to neovascular AMD	23%
Linear [66]	One or more hyperautofluorescent linear lesions	Hyperpigmentation	Slow progression	3%
Lace-like [66]	Network of branching hyperautofluorescent lines	Hyperpigmentation	Slow progression	2%
Reticular [66]	Multiple small dots of hypoautofluorescence	Reticular pseudodrusen in the supero-temporal quadrant	Documented progression to exudative changes	15%
Speckled [66]	Irregular abnormalities extending beyond the macular area with mottled aspect	Pigment abnormalities +/− soft confluent drusen	Slow progression	26%
Focal-plaque-like [86]	Large hyperautofluorescent region	Hyperpigmentation +/− soft drusen	Moderate risk of progression	Not available

**Table 4:** FAF junctional zone patterns in GA [19,67].

FAF Pattern	Frequency	Junctional Zone Pattern	Clinical Correlations [19]
None [67]	12.1%	No abnormal FAF junctional zone	Slowest progression 0.38 mm^2^/year
Focal [67]	12.1%	Small hyper-FAF dots at lesion margins	Reduced progression 0.81 mm^2^/year
Banded [67]	12.8%	Perilesional band of hyper-FAF at the GA margin	Rapid progression 1.81 mm^2^/year
Patchy [67]	2%	Irregular large area of hyper-FAF	Fast progression 1.84 mm^2^/year
Diffuse	57%		Mean progression rate 1.77 mm^2^/year
- Branching [67]	27.5%	Hyper-FAF branches at the GA margin	
- Reticular [67]	4.7%	Hyper-FAF radial lines	
- Fine granular [67]	18%	Large area of hyper-FAF with small particles around the GA	
- Fine granular with peripheral punctate spots [67]	18.1%	Hyper-FAF with extended lesions around the GA	
- Trickling [19]	N/A	Grey spreading central zone with hyper-FAF margins	Very fast progression 3.02 mm^2^/year

**Table 5:** Complementary roles of FAF and OCT in the clinical assessment of AMD.

Clinical Parameter/Lesion	OCT	FAF
RPE/Photoreceptor dysfunction/degeneration	Structural lesions—thinning or disruption of ellipsoid zone [72]	Hyper/hypo-autofluorescence [72]
Retinal fluorophore distribution	-	Generates a topographic representation of lipofuscin distribution [28] 24 June 2025 10:14:00 a.m.
Drusen	Size, homogeneity, shape, reflectivity, ellipsoid zone status characterization [72]	Autofluorescence appearance modified mainly by drusen size and ellipsoid zone [72]
	Elevations of RPE/Bruch’s membrane complex [74] 24 June 2025 10:14:00 a.m.	Normal/increased/decreased autofluorescence [74]
SDD	Hyper-reflective deposits above the RPE [76]	Hypo-autofluorescent lesions [76]
GA	RPE loss >250 microns, ellipsoid zone disruption, increased choroidal transmission >250 microns, outer nuclear layer thinning [107]	Hypoautofluorescent area >250 microns [107]
	Incipient detection of lesions, structural analysis	Follow-up of established GA [107]
RPE tear	RPE dehiscence adjacent to an elevated PED, with the RPE appearing retracted and irregular [140]	RPE tear appears as central hypoautofluorescence due to exposed choroid, with adjacent hyperautofluorescence at the retracted RPE edge [141]
nAMD	Incipient CNV	Normal FAF [68,70]
	Type 1: CNV under the RPE	Irregular pattern of FAF [68]
	Type 2: CNV above the RPE	Hypoautofluorescent lesion [68]
	Disciform scar/subretinal hyper-reflective material [142]	Mostly reduced FAF [37]

**Table 6:** Comparative analysis of B-FAF vs. G-FAF imaging in AMD: Key study findings.

Study	Device	Keypoints
Wolf-Schnurrbusch et al. (2011) [57]	HRA platform (Heidelberg Engineering); B-FAF: 488 nm, G-FAF: 514 nm	Macular pigment absorption in B-FAF led to diminished foveal autofluorescence, which may obscure central atrophy. B-FAF may falsely imply foveal involvement and overestimate the GA size. G-FAF provided better delineation of lesion borders, especially in the central/foveal region. Conclusions: G-FAF is superior for assessing small, central lesions, and GA quantification, while B-FAF performs well in the periphery.
Pfau et al. (2017) [122]	HRA2/Spectralis (Heidelberg Engineering); B-FAF: excitation 488 nm, emission 500–700 nm, G-FAF: 518 nm	B-FAF provided good lesion visualization, but macular pigment interference reduced reproducibility. G-FAF showed highest inter-reader agreement and more consistent morphological lesion characteristics. Conclusions: G-FAF is more reproducible, making it preferable for use in clinical trials requiring precise grading.
Corradetti et al. (2022) [118]	SLO Mirante (Nidek); B-FAF: 488 nm, G-FAF: 532 nm	B-FAF served as a standard imaging tool with high contrast for GA detection. G-FAF offered comparable accuracy, slightly more patient-friendly, and less foveal shadowing. Conclusions: B-FAF and G-FAF were equivalent in GA measurement; both can be used reliably in practice.
Froines et al. (2024) [125]	B-FAF: Spectralis (488 nm excitation, 500 nm barrier filter), G-FAF: Optos Ultrawidefield (532 nm, 633 nm excitation, 540 nm barrier filter)	B-FAF reported larger GA areas. UWF G-FAF underestimated GA size. G-FAF offered clearer foveal visualization, aiding the measurement of GA proximity to the foveal center. Conclusions: Both modalities showed similar GA progression rates. Differences in imaging systems suggest that consistency is essential in longitudinal follow-up.
Abbasgholizadeh et al. (2024) [127]	B-FAF: Spectralis HRA + OCT2 (488 nm), G-FAF: Optos Ultrawidefield (532 nm)	B-FAF enabled semiautomated GA quantification using Region Finder with high segmentation precision. G-FAF provided ultrawidefield coverage and strong correlation with B-FAF, but GA sizes differed. Conclusions: Both are reliable, but not interchangeable over time. Use the same system consistently in follow-up.

**Table 7:** Multimodal imaging platforms.

Imaging Platform	OCT Type	FAF Excitation Wavelength	Confocal SLO	FA/ICGA	Distinctive Additional Features
Heidelberg Spectralis [143,144] (Heidelberg Engineering, Heidelberg, Germany)	SD	488 nm (Blue)	Yes	Optional	Widely used in research and clinical trials
Topcon DRI OCT Triton Plus [145,146] (Topcon, Inc., Tokyo, Japan)	SS	535–585 nm (Green)	Yes	Yes	Spaide filters
Optos Silverstone [147,148] (Optos PLC, Dunfermline, Scotland, United Kingdom)	SS	488 nm/532 nm (Blue/Green)	Yes	Yes	Ultra-widefield view
Nidek Mirante [149] (Nidek Co., Gamagori, Japan)	SD	488 nm/532 nm (Blue/Green)	Yes	Yes	Retromode Imaging

## Data Availability

Not applicable.
